# Online Obsessive-Compulsive Disorder Treatment: Preliminary Results of the “OCD? Not Me!” Self-Guided Internet-Based Cognitive Behavioral Therapy Program for Young People

**DOI:** 10.2196/mental.5363

**Published:** 2016-07-05

**Authors:** Clare Samantha Rees, Rebecca Anne Anderson, Robert Thomas Kane, Amy Louise Finlay-Jones

**Affiliations:** ^1^School of Psychology and Speech PathologyFaculty of Health SciencesCurtin UniversityPerthAustralia

**Keywords:** adolescent, anxiety disorders/therapy, Australia, Internet, obsessive-compulsive disorder, self-care, therapy, computer-assisted/statistics and numerical data, treatment outcome, young adult, iCBT, adolescents

## Abstract

**Background:**

The development and evaluation of Internet-delivered cognitive behavioral therapy (iCBT) interventions provides a potential solution for current limitations in the acceptability, availability, and accessibility of mental health care for young people with obsessive-compulsive disorder (OCD). Preliminary results support the effectiveness of therapist-assisted iCBT for young people with OCD; however, no previous studies have examined the effectiveness of completely self-guided iCBT for OCD in young people.

**Objective:**

We aimed to conduct a preliminary evaluation of the effectiveness of the OCD? Not Me! program for reducing OCD-related psychopathology in young people (12-18 years). This program is an eight-stage, completely self-guided iCBT treatment for OCD, which is based on exposure and response prevention.

**Methods:**

These data were early and preliminary results of a longer study in which an open trial design is being used to evaluate the effectiveness of the OCD? Not Me! program. Participants were required to have at least subclinical levels of OCD to be offered the online program. Participants with moderate-high suicide/self-harm risk or symptoms of eating disorder or psychosis were not offered the program. OCD symptoms and severity were measured at pre- and posttest, and at the beginning of each stage of the program. Data was analyzed using generalized linear mixed models.

**Results:**

A total of 334 people were screened for inclusion in the study, with 132 participants aged 12 to 18 years providing data for the final analysis. Participants showed significant reductions in OCD symptoms (*P*<.001) and severity (*P*<.001) between pre- and posttest.

**Conclusions:**

These preliminary results suggest that fully automated iCBT holds promise as a way of increasing access to treatment for young people with OCD; however, further research needs to be conducted to replicate the results and to determine the feasibility of the program.

**Trial Registration:**

Australian New Zealand Clinical Trials Registry (ANZCTR): ACTRN12613000152729; https://www.anzctr.org.au/Trial/Registration/TrialReview.aspx?id=363654 (Archived by WebCite at http://www.webcitation.org/ 6iD7EDFqH)

## Introduction

Obsessive-compulsive disorder (OCD) is a potentially disabling psychological condition affecting 0.5% to 3% of children and adolescents [1-4]. The disorder is associated with high levels of comorbidity [1,5] and significant psychosocial impairment [6,7], such as difficulties concentrating at school and completing homework, disruption in household routines, and impairments in social functioning [6]. When young people with OCD do not receive adequate treatment, they are at risk of experiencing continued symptoms and additional psychopathology in adulthood [8-10]. As a result, early intervention is imperative.

The development of Internet-based cognitive behavioral therapy (iCBT) for OCD provides a promising pathway toward increasing the accessibility and availability of evidence-based treatment for young people with OCD. Substantial evidence supports the effectiveness of face-to-face cognitive behavioral therapy (CBT) with exposure and response prevention (ERP) as the gold-standard psychotherapeutic intervention for OCD [11,12]. However, there are significant limitations to the availability and accessibility of this treatment in the community [13-16]. In addition, young people may be unlikely to seek treatment for anxiety disorders, thereby increasing the likelihood of long-term impairment [17,18]. Online treatments help to overcome these obstacles by providing immediate, cost-effective, and remote access to treatment [19], and emerging evidence supports the use of iCBT and computerized CBT (cCBT) in young people with depression and anxiety. For example, one meta-analysis of 13 randomized controlled trials found moderate-to-large effect sizes across studies of iCBT and cCBT in the treatment of depression and anxiety in children and adolescents [20]. Another meta-analysis found that the effectiveness of iCBT for childhood anxiety is comparable to that of face-to-face CBT [21], whereas in a systematic review, Richardson et al [22] found that young people and their parents report moderate-to-high levels of satisfaction with cCBT for depression and anxiety, although attrition and noncompletion rates are often high.

Although research on the development and evaluation of Internet-based treatment for children and adolescents with OCD is relatively limited, these results suggest that iCBT may be a viable intervention for young people with this disorder. To our knowledge, only one study has examined the efficacy of iCBT for OCD in young people [23]. Using an open trial, Lenhard et al [23] delivered a 12-week therapist-assisted iCBT program to 21 young people aged 12 to 17 years who had a primary diagnosis of OCD. Participants reported significant pre-post reductions in OCD severity and related impairment, as well as significant improvements on measures of anxiety and global functioning. Although limited by the small sample size, these results are encouraging and raise the question of whether similar outcomes might be achieved without the inclusion of therapist support.

The minimal costs and consistent availability and accessibility associated with self-guided online programs supports the proposition that iCBT without therapist assistance has the potential to confer important public health benefits, if deemed safe and effective [19]. Moreover, such programs may be preferable for individuals who are concerned about stigma, confidentiality, or talking to a therapist about personal issues [24,25]. Preliminary results from studies with adults support the efficacy of self-guided iCBT for OCD [26], with initial evidence suggesting long-term (12-month) impact [24]. However, a recent meta-analysis suggests that completely self-guided interventions for adults with OCD suffer from high attrition rates, and that therapist-assisted iCBT produces superior treatment outcomes [27]. Across studies of iCBT for other anxiety and mood disorders, it has been found that when dropout and compliance is taken into account, therapist-assisted iCBT is more effective than self-guided iCBT [28]. An important question that remains to be answered is whether purely self-guided iCBT is effective for young people with OCD.

### Aims and Hypotheses

To our knowledge, there is no prior research evaluating the impact of fully self-guided iCBT for young people with OCD. Therefore, we aimed to conduct a preliminary evaluation to determine whether self-guided iCBT using the “OCD? Not Me!” program was effective in reducing OCD psychopathology in young people. We formed two main hypotheses: (1) that mean number of OCD symptoms would significantly decrease between pre- and posttest and (2) that mean OCD severity would significantly decrease between pre- and posttest. We also aimed to investigate the pattern of change in OCD psychopathology over time in the program.

## Methods

### Study Design and Procedures

The data for this study were early and preliminary data collected as part of a longer study currently being conducted to investigate the effectiveness and feasibility of the OCD? Not Me! program for reducing OCD symptoms and related distress among young people with OCD [29]. This study is a 4-year open trial utilizing a within-groups design to examine pre-post changes in young people’s OCD symptoms and severity, associated functional impairment, quality of life, family accommodation, and self-esteem, as well as parent/caregiver distress. The study was approved by the Curtin University Human Research Ethics Committee, Bentley, Western Australia, Australia (HR 45/2013).

### Intervention

**Figure 1:**
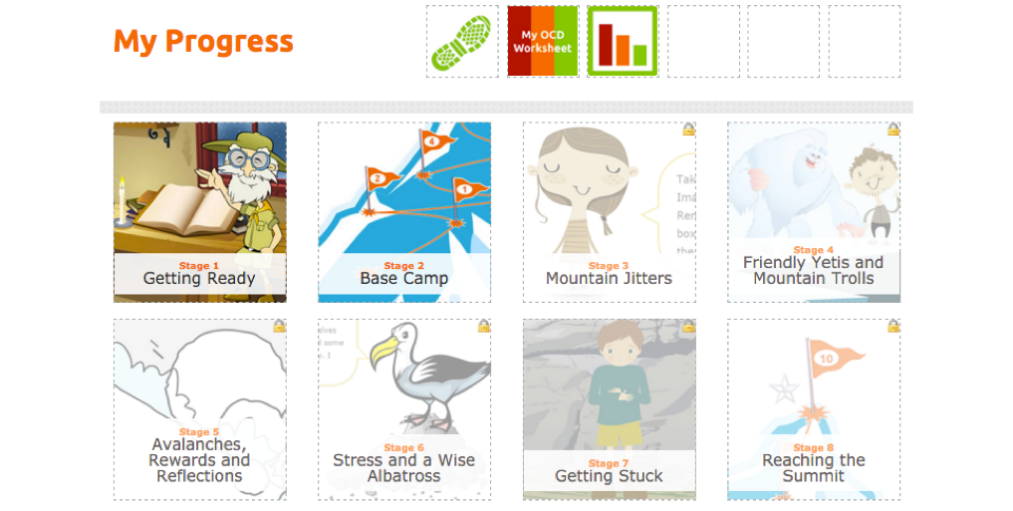
Screenshot from the OCD? Not Me! program: overview of the eight stages of the program.

**Figure 2:**
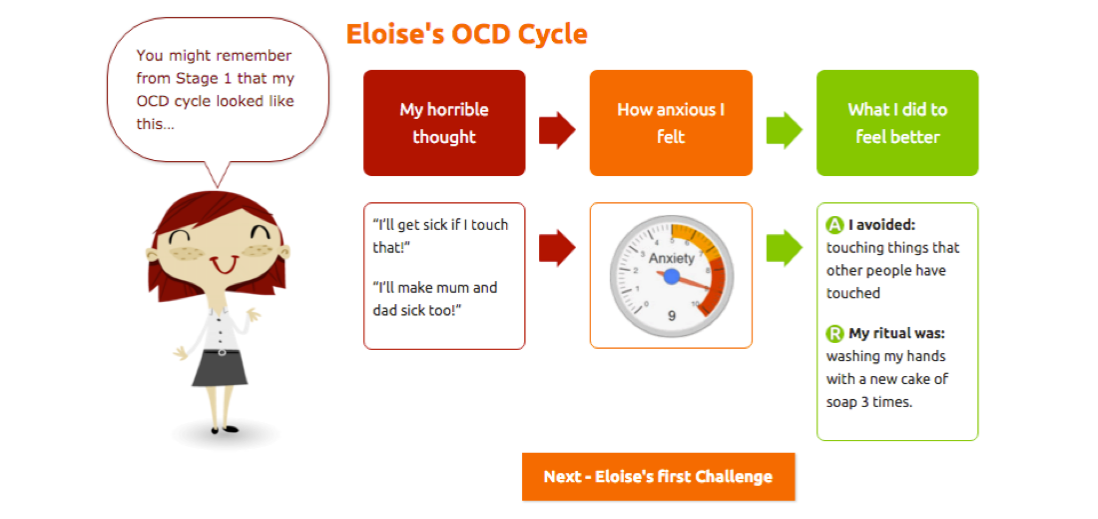
Screenshot from the OCD? Not Me! program: illustration of the OCD cycle explaining the functional link between obsessions and compulsions.

**Figure 3:**
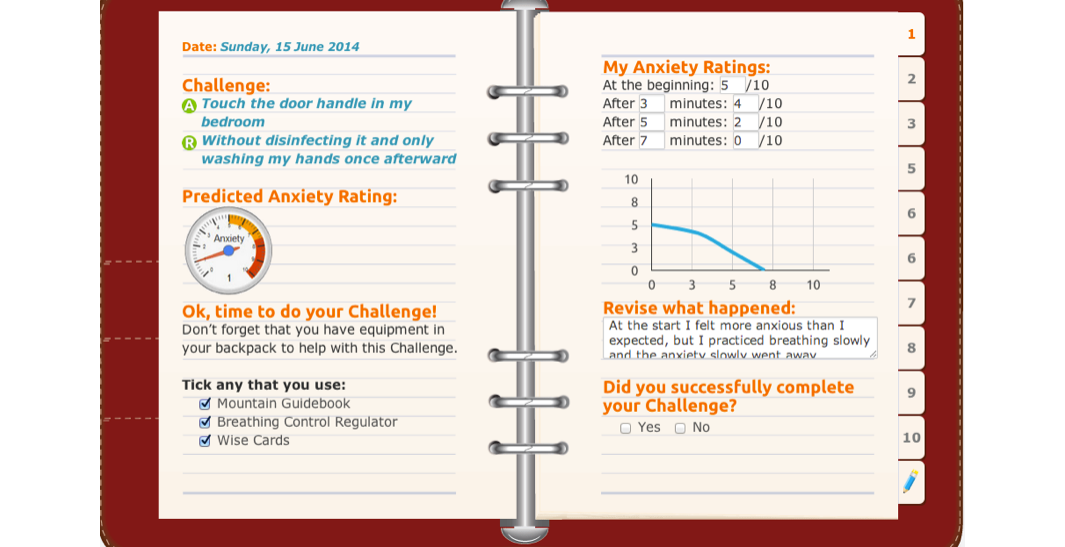
Screenshot from the OCD? Not Me! program: the log book used to record OCD Mountain Challenges (ERP exercises).

As described in Rees et al [29], OCD? Not Me! is a fully automated, eight-stage online program that is designed to treat symptoms of OCD in young people aged 12 to 18 years (for an overview of the eight stages of the program, see Figure 1). The treatment protocol is structured around the metaphor of “climbing OCD Mountain” to conquer OCD symptoms; in undertaking this journey, young people are provided with psychoeducation regarding OCD and a rationale for treatment using CBT with ERP. They are taught how to identify the functional link between their obsessions and compulsions (Figure 2), how to construct exposure exercises to target their OCD symptoms, and how to construct an exposure hierarchy that will support them to reduce their compulsions in a gradual way. In this process, participants in the program learn strategies for habituating to anxiety, dealing with problematic cognitions, and managing stress and setbacks. The strategies provided in the program are illustrated using the metaphor of “mountaineering equipment” that supports the participant to ascend OCD Mountain, and include an interactive log book for participants to record their OCD Mountain Challenges (ERP exercises) as they complete them (see Figure 3). At each stage of the program, parents and caregivers are also emailed with a link to online resources. These online resources outline information about what the young person is learning in the program, provide tips for supporting the young person in the program, and help parents and caregivers to manage family/caregiver stress.

### Participant Eligibility and Recruitment

The OCD? Not Me! program is designed for youth aged 12 to 18 years; although individuals outside this age bracket are allowed to access the program, their data were not used in this study. Because the OCD? Not Me! program is designed to treat OCD, to be eligible for inclusion in the program, potential participants were required to have at least subclinical symptoms of OCD. The Short OCD Screener (SOCS) [30] was used to screen for OCD symptoms, with participants required to have a SOCS score of two or more to be included in the study. We expected that young people with eating disorders might be drawn to the program, given that rapid weight loss and starvation can cause obsessional thinking [31]. Our online measures were not able to differentially diagnose eating disorders from OCD to the degree that a clinician was able and, given the potential need for more intensive and specialized treatment for individuals with eating disorder symptoms, participants were excluded from the study if they reported symptoms of an eating disorder. The SCOFF questionnaire was used to screen for these criteria [32], with participants excluded if they reported a score of two or more on this measure. Similarly, due to the overlap between psychosis symptoms and psychotic-like symptoms in OCD [33], participants were excluded from the program if they reported moderate-to-high symptoms of psychosis. The Adolescent Psychotic-Like Symptom Screener (APSS) [34] was used to screen for this outcome, and participants were excluded from the program if they reported a score of four or more on this measure. Finally, participants who reported moderate-to-high suicide risk were excluded from the study and encouraged to seek more specialized services. To assess suicide and self-harm risk, a measure was adapted from the suicide risk assessment module of the Mini-International Neuropsychiatric Interview for Children and Adolescents (MINI-KID) [35].

Participants were self-referred or recommended the program by a friend, family member, or health care provider. Written consent to participate in the study was obtained from all participants and their parents/caregivers online. All screening measures were completed online, and individuals who were not eligible for participation in the program were referred to the treatment provider database on the OCD? Not Me! website. Using this website, individuals can search for face-to-face mental health services in their local area, as well as access phone numbers for crisis counseling hotlines. Participants who were deemed eligible for participation in the program went on to complete the online assessment measures detailed subsequently.

### Measures

#### Demographic Measures

Participants were asked to complete a series of demographic questions regarding their gender, age, country and state of residence, and education. Participants were also asked whether they were currently receiving psychological treatment for OCD and whether they were currently prescribed medication for OCD.

#### Obsessive-Compulsive Disorder Diagnosis and Comorbid Diagnoses

There were no freely available, online, self-report comprehensive psychiatric assessments for young people; therefore, we developed the Youth Online Diagnostic Assessment (YODA) to evaluate whether participants met diagnostic criteria for OCD as outlined in the fifth edition of the *Diagnostic and Statistical Manual of Mental Disorders* (*DSM-V*) [36]. The YODA covers the majority of disorders outlined in the *DSM-V*, although only the OCD diagnostic section was used in this study.

#### Obsessive-Compulsive Disorder Symptoms and Severity

The self-report version of the Children’s Florida Obsessive-Compulsive Inventory (C-FOCI) [37] was used to assess OCD symptoms and severity. The C-FOCI is a brief measure of OCD psychopathology in children and adolescents that consists of a 17-item symptom checklist, and a five-item severity scale. The C-FOCI has been validated for Internet administration and has adequate psychometric properties [37]. In this sample, the internal consistency for the symptoms scale was α=.79, whereas the internal consistency for the severity scale was α=.82.

#### Procedure

Following screening, participants completed pretest measures online and were given access to the program once pretest measures were complete. Although the recommended time frame for the program is 8 weeks, participants completed the program at their own pace. At the beginning of each stage of the program, participants were asked to complete a brief online assessment consisting of the C-FOCI and a risk assessment for suicide and self-harm. Therefore, C-FOCI data were collected at nine time points throughout the study: pretest, posttest, and at the beginning of stages 2 to 8 of the program.

### Analysis

Data were analyzed with generalized linear mixed models (GLMM) using the GENLINMIXED procedure of SPSS (version 22.0), with “participant” treated as a random effect and “stage” (pretest, per stage 2-8, and posttest) treated as a fixed effect. To accommodate violations of sphericity, the covariance matrix was changed from the default of compound symmetry to autoregressive (ARMA11). To maximize the likelihood of convergence, separate GLMM analyses were conducted for both of the outcome variables, and alpha levels were corrected to control for inflation of the family-wise error rate. The Bonferroni-corrected alpha level for all statistical tests was .025.

The GLMM maximum likelihood procedure is a full information estimation procedure that uses all the data available at each assessment point, rather than requiring data from all participants at each point. This optimizes statistical power and reduces sampling bias associated with subject attrition [38,39], making it suitable for research on Internet-based interventions, which often demonstrate high dropout rates [40]. Additionally, this analysis is robust to unevenly distributed assessment points [39,41]. Two GLMMs were used to evaluate the relationship between the fixed effect of time and each of the subscales of the C-FOCI (symptoms and severity). Post hoc least significant difference (LSD) tests were conducted to test for significant differences between time points. The *t* test values for the T1-T9 effects were computed using maximum likelihood (ML) to compensate for missing data. Cohen’s *d* [42] calculations were conducted to determine the size of pre-posttest change. Effect size magnitude was interpreted using Cohen’s [42] conventions (0.2=small, 0.5=moderate; and ≥0.8=large).

## Results

**Figure 4:**
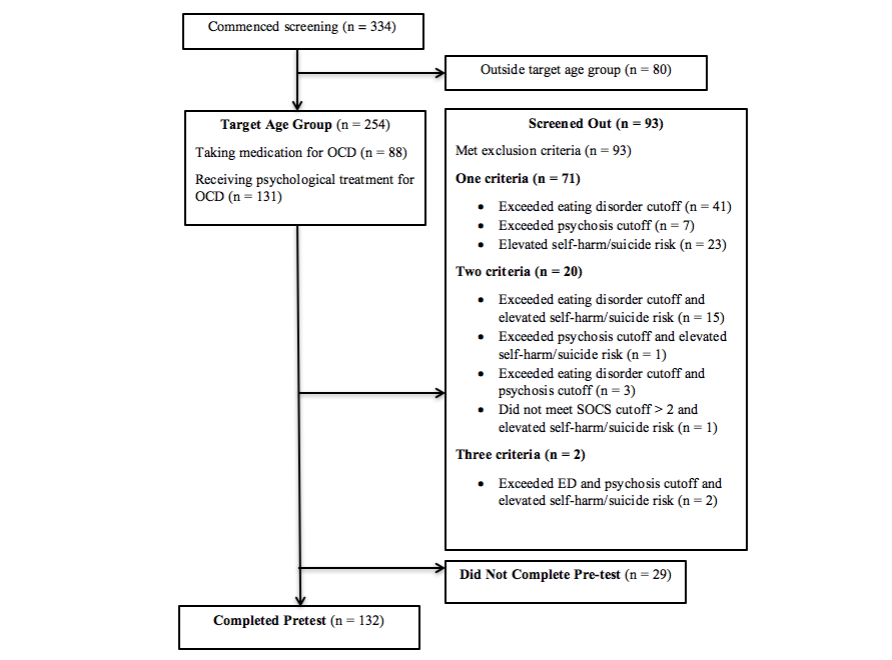
Flowchart of assessment and clinical characteristics of screened participants.

Of the 334 potential participants who completed screening measures, 21 were younger than 12 years and 59 were older than 18 years. Their data were not included in these analyses. Of the 254 participants were within the target age range, 93 participants were screened out because they met the exclusion criteria. A further 29 participants failed to complete pretest assessments. The screening and pretest procedure and number of participants meeting each exclusion criteria are detailed in Figure 4.

A total of 132 participants who were in the target age range completed pretest measures. This sample was 43.2% (57/132) male and 56.8% (75/132) female, with a mean age of 14.58 years (SD 1.94). In this sample, 73.5% (97/132) of participants met the *DSM-V* criteria for OCD as determined using the YODA.

### Participant Flow

The number of participants that commenced each stage of the program at the time the data were collected were: stage 1 (n=116), stage 2 (n=67), stage 3 (n=27), stage 4 (n=16), stage 5 (n=14), stage 6 (n=12), stage 7 (n=11), and stage 8 (n=11).

### Estimated Means

Estimated means were used to describe the average pretest scores on the C-FOCI for the target sample. The pretest (T1) mean score for OCD symptoms was 7.82 (SE 0.34) and 11.56 (SE 0.31) for OCD severity. At posttest (T9), the means for these outcomes were 3.87 (SE 0.83) and 5.77 (SE 0.97), respectively. Per-stage means for each outcome are shown in Table 1.

### Main Effects of Time and Pairwise Contrasts

Our first hypothesis predicted a significant main effect of time on mean number of OCD symptoms, using a Bonferroni-adjusted alpha level of .025. This hypothesis was supported (*F*_8,285_=7.38, *P<*.001), with significant decrease in OCD symptoms observed between pre- and posttest (*P*<.001). Per-stage LSD tests with pairwise contrasts indicated that significant decreases in OCD symptoms occurred between stages 1 and 2, stages 2 and 3, stages 3 and 4, and stages 6 and 7 (Table 2). A slight, nonsignificant increase in OCD symptoms was reported between stage 8 and posttest. Effect size calculations indicated a moderate effect for the changes in OCD symptoms between pre- and posttest (*d=*.64).

**Table 1:** Estimated means and standard errors for OCD symptom and severity scores.

Outcome	Test time, mean (SE)								
	T1	T2	T3	T4	T5	T6	T7	T8	T9
Symptoms	7.82 (0.34)	7.34 (0.35)	6.76 (0.44)	5.93 (0.52)	5.72 (0.60)	5.33 (0.62)	4.30 (0.69)	3.76 (0.82)	3.87 (0.83)
Severity	11.56 (0.31)	10.61 (0.43)	10.40 (0.54)	9.52 (0.88)	8.60 (0.88)	8.10 (0.96)	6.92 (0.95)	5.99 (1.18)	5.77 (0.97)

**Table 2:** Least significant difference (LSD) tests of the simple main effects of time with pairwise contrasts (contrast estimate; CE) of OCD symptoms and severity (T1-T9).

Outcome and time interval		CE (SE)	*t*_285_	95% CI	*P* (adjusted)
**Symptoms**					
	T1-T9	3.96 (0.84)	4.70	2.30, 5.61	<.001
	T1-T2	0.49 (0.15)	3.26	0.19, 0.78	.001
	T2-T3	0.58 (0.27)	2.15	0.05, 1.11	.003
	T3-T4	0.82 (0.25)	3.24	0.32, 1.33	.001
	T4-T5	0.21 (0.27)	0.79	–0.32, 0.74	.43
	T5-T6	0.39 (0.34)	1.15	–0.27, 1.05	.25
	T6-T7	1.03 (0.38)	2.75	0.29, 1.77	.006
	T7-T8	0.54 (0.35)	1.53	–0.15, 1.22	.13
	T8-T9	–0.10 (0.37)	–0.28	–0.83, 0.62	.78
**Severity**					
	T1-T9	5.79 (0.99)	5.87	3.85, 7.73	<.001
	T1-T2	0.96 (0.34)	2.79	0.28, 1.63	.006
	T2-T3	0.21 (0.52)	0.41	–0.81, 1.24	.68
	T3-T4	0.88 (0.63)	1.38	–0.37, 2.12	.17
	T4-T5	0.91 (0.58)	1.57	–0.23, 2.05	.12
	T5-T6	0.50 (0.34)	1.45	–0.18, 1.18	.15
	T6-T7	1.18 (0.35)	3.37	–0.49, 1.86	.001
	T7-T8	0.94 (0.41)	2.28	0.13, 1.74	.02
	T8-T9	0.22 (0.65)	0.34	–1.05, 1.49	.73

Our second hypothesis predicted a significant main effect of time on mean OCD severity, using a Bonferroni-adjusted alpha level of .025. This hypothesis was also supported (*F*_8,285_=15.21, *P<*.001), with significant decreases in OCD symptom severity observed between pre- and posttest (*P*<.001). Per-stage LSD tests with pairwise contrasts indicated that significant decreases in OCD severity occurred between stages 1 and 2, stages 6 and 7, and stages 7 and 8. Although OCD severity continued to decrease steadily over the course of the program, other per-stage reductions did not reach significance (Table 2). Effect size calculations indicated a large effect for the changes in OCD severity between pre- and posttest (*d=*.89).

## Discussion

This study aimed to evaluate early and preliminary results from a longer trial examining the impact of the OCD? Not Me! program on OCD symptoms and severity in young people aged 12 to 18 years. This is the first study to examine the effectiveness of a fully automated iCBT program for reducing OCD symptomology in young people.

Results from this study are encouraging. Participants in this study reported average pretest OCD symptoms and severity that were higher than those reported by a sample of young people with a primary diagnosis of OCD (mean 6.22, SD 3.54 for OCD symptoms and mean 11.22, SD 4.07 for OCD severity) in Storch et al’s [37] study. Although assessment of OCD symptoms in this study was based on self-report rather than diagnostic interview, it appears that, on average, participants were experiencing levels of OCD pathology comparable with clinical samples. Significant decreases in OCD symptoms and severity were observed between pre- and posttest. Per-stage analyses of OCD symptoms and severity indicated that mean scores on these outcomes decreased gradually over time in the program. In addition, effect size calculations demonstrated a moderate effect for reduction in OCD symptoms and a large effect for reduction in OCD severity. These effect sizes may be contrasted with Lenhard et al [23], who reported large effect sizes for reduction in OCD symptoms and severity (*d=* 1.09 and *d=* 1.43, respectively) in their trial of therapist-assisted iCBT for adolescents with OCD, although it should be noted that they used a different measure to evaluate OCD symptoms and severity. A recent meta-analysis reported standardized effect sizes of 0.70 across five studies of iCBT with varying levels of therapist assistance for childhood anxiety [21]. Importantly, it should be emphasized that our results were observed in the absence of any therapist involvement. This is an exciting finding given the notable limitations in the accessibility and availability of evidence-based psychotherapeutic treatment for young people with OCD. As Klein et al [43] have pointed out, the availability of fully automated, effective e-mental health interventions means that people can access treatment immediately and remotely, at a time and location that suits them.

It should be highlighted that these results are preliminary, and the final results of the trial might be quite different. Additionally, although entirely self-help programs make an important contribution to stepped care approaches to mental health, the value of some level of therapist contact should not be dismissed. A recent meta-analysis of self-help interventions for adults with OCD found that across studies, attrition rates declined and clinical outcomes improved with increasing therapist contact [27]. It would be beneficial for future research to consider how the inclusion of therapist assistance might impact outcomes and completion rates in the OCD? Not Me program. Future research is also required to determine those young people fully self-guided programs are appropriate for, and those from whom therapist contact is necessary.

Because the OCD? Not Me! program (including assessment) is designed to be entirely self-guided, this study did not use therapist-administered clinical interviews and, therefore, it cannot be determined whether participation in the program made an impact on OCD as a diagnosis. As assessment must be conducted without therapist involvement in order for self-guided programs to be truly self-help and anonymous, a key direction for future research is the development and evaluation of online, self-administered diagnostic interviews. We recommend further research to determine the validity, sensitivity, and specificity of the YODA relative to face-to-face diagnostic assessment.

A further limitation of this study was because it was an open trial with no control group, it cannot be reliably concluded that changes in the outcome measures were due to participation in the intervention. However, it should be noted that OCD in childhood and adolescence is characterized as a chronic disorder with high persistence rates [8,44]; therefore, it is unlikely that the observed outcomes are linked to spontaneous remission. It is recommended that future research compare the efficacy of the OCD? Not Me! program to a waitlist control group in order to more reliably link outcomes to the effects of the intervention. It is also recommended that future research on this intervention include some follow-up assessment to investigate whether changes in symptomology are durable over time.

Attrition analyses were not conducted in the current study because the study is ongoing and some participants were still undergoing treatment at the time of collecting this data. Although the GLMM procedure is particularly useful for conducting intention-to-treat analyses in studies where moderate-high rates of attrition are anticipated [39], attrition analyses are recommended in future studies. Understanding the factors that predict attrition (including attrition at pretest) is essential to understanding who fully automated iCBT is most suitable for and how the program might be adjusted to optimize participant retention.

It should be noted that almost 25% of the sample who completed screening measures in this study were outside of the intended age range for the iCBT program, indicating that there is a need for online OCD treatment services that target children younger than 12 years, as well as adults older than 18 years. In addition, almost a third of potential participants in the target age range were screened out of the study for meeting one or more of the exclusion criteria (elevated eating disorder or psychosis symptoms, and/or elevated suicide/self-harm risk). There is a clear need for services that are targeted to this group, and for better understanding of how best to manage risk within the context of self-guided treatment. Although most efficacy studies exclude individuals with suicide or self-harm risk, it is recommended that future developments in iCBT consider including strategies and protocols for managing suicide and self-harm risk, to support translation into effective intervention.

One of the benefits of the OCD? Not Me! program is that it includes a searchable database of mental health providers that participants can use to seek more intensive or specialized services, such as face-to-face treatment or telephone support services. Our data indicate that such services are relevant for those who seek treatment via the online program (who are screened out or who require more intensive services due to the severity of their symptoms). In addition, the findings support the potential for online assessments and services to be more comprehensively integrated into stepped care models, with treatment-seekers offered the most appropriate level of care for their needs following assessment. Increasing communication between online platforms and frontline health services supports continuity of care and also reduces the need for repeated assessments by different health care professionals. On this point, we found that approximately half the participants in our sample were already receiving psychotherapeutic treatment for OCD, and a third were currently taking prescribed medication for the disorder. This finding suggests that it may be of benefit to support health practitioners to use the iCBT programs with their clients through the provision of manuals and training.

In conclusion, the results of this study provide preliminary evidence that fully automated iCBT offers promise as a way of increasing access to effective treatment for young people with OCD. Future research is needed to replicate these results and to investigate predictors of treatment retention and outcome.

## Abbreviations

CBT: cognitive behavioral therapy
CE: contrast estimate
ERP: exposure and response prevention
GLMM: generalized linear mixed models
LSD: least significant difference
ML: maximum likelihood
OCD: obsessive-compulsive disorder
SOCS: Short OCD Screener
YODA: Youth Online Diagnostic Assessment
